# Molecular Cartography: Mapping the Landscape of Meiotic Recombination

**DOI:** 10.1371/journal.pbio.0050333

**Published:** 2007-12-18

**Authors:** Jing Pan, Scott Keeney

## Abstract

Mapping recombination hot and cold spots in yeast has previously relied on mutants, which themselves distort the map. Now a new method promises to overcome that problem.

***Hic sunt dracones***

*–“Here be dragons.” Text popularly thought to appear on ancient maps to indicate unexplored or dangerous lands, although the only known such instance of the phrase appears on the Lenox Globe, ∼1500–1510 (collection of the New York Public Library) [1]*.

Meiosis is the specialized cell division that gives rise to gametes (reproductive cells such as sperm or eggs, or spores in yeast). To complete meiosis, a cell duplicates its chromosomes once but then goes through two rounds of chromosome segregation, reducing the genome by half. The original chromosome number is restored by the fusion of gametes during fertilization to form a zygote. Meiosis is a fundamental bottleneck passed by each generation in a sexually reproducing organism (Figure 1).

**Figure 1:**
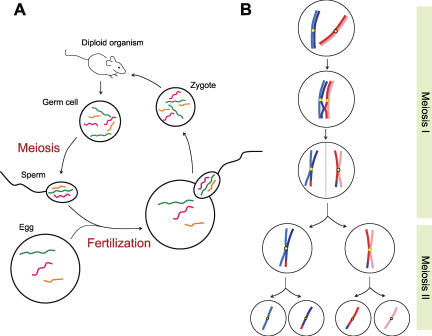
Chromosome Cycle of Meiosis and Sexual Reproduction (A) Simplified reproductive cycle of sexual organisms. Chromosomes are represented as colored lines, with two of each color to represent the maternal and paternal copies of each. During meiosis, the number of chromosomes is reduced by half to generate gametes (e.g., sperm and eggs). Diploidy is restored upon fertilization. (B) Two consecutive cell divisions in meiosis. In meiosis I, homologous chromosomes recombine and exchange genetic information before they separate from one another. In meiosis II, sister chromatids separate.

In the first meiotic division, homologous maternal and paternal chromosomes are separated, whereas sister chromatids—the pair resulting from chromosome replication prior to meiosis—are separate in the second division [2] (Figure 1B). During meiosis in most organisms, genetic information is exchanged between chromosomes through homologous recombination, which serves important biological functions. At the genomic level, recombination creates diversity by reshuffling genetic information between homologous chromosomes. At the cellular level, recombination generates temporary connections between homologous chromosomes that are required for accurate segregation [3]. Alterations in the normal recombination pattern are often associated with errors in chromosome segregation in humans, and these errors are a major cause of spontaneous abortions and congenital birth defects, including mental retardation [4].

## Meiotic Recombination Does Not Occur at Random Throughout the Genome

**Figure 2:**
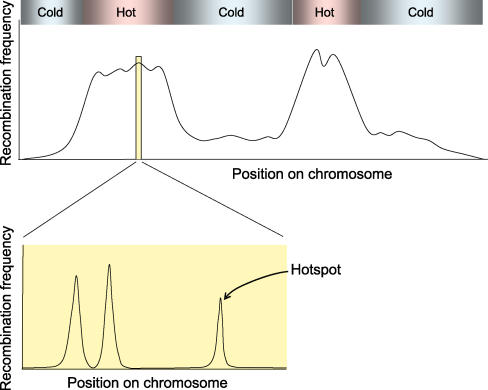
Recombination Frequency Mapped at Different Resolutions The upper plot is a highly schematic representation of recombination distribution along an entire chromosome, showing alternating domains of relatively higher (“hot”) and relatively lower (“cold”) recombination activity. Such domains are megabase pairs long in mammals and 20–100 kb long in yeast. When examined at higher resolution, distinct hot spots are revealed, separated by DNA segments where little, if any, recombination occurs (lower plot). Recombination hot spots in mammals are typically ∼1–2 kb wide, separated by tens of kilobases of recombinationally suppressed DNA. For examples of real recombination distributions, see [6,25,26].

In most organisms where sufficient information is available, each chromosome can be divided into large regions of relatively high or low recombination rates (“hot” and “cold” domains, respectively) [5, 6] (Figure 2A). Within these domains, most recombination occurs within small regions called “hot spots” (typically 1–2 kilobases [kb] wide in humans and mice) that are separated by larger stretches of DNA where recombination occurs rarely, if at all [7] (Figure 2B). (Note that cold domains are not devoid of hot spots—instead, the aggregate recombination activity of all hot spots in a domain is lower for cold domains than for hot ones.)

Deriving accurate maps of the peaks and valleys that make up the recombination landscape is of interest for several reasons. First, understanding where recombination happens sheds light on the evolution of genome structure. Second, such maps help to reveal mechanisms behind this uneven distribution. Finally, knowledge of human recombination distribution facilitates association studies to identify disease-related genes.

Here we focus on efforts to understand recombination distributions in the budding yeast Saccharomyces cerevisiae. Yeast is an outstanding organism that allows researchers to work out molecular details of recombination, because DNA intermediates in the pathway can be analyzed directly, it lends itself to easy genetic manipulation, and most of the proteins involved are evolutionarily conserved.

## Hot Spots Are Preferential Sites for DNA Double-Strand Breaks

**Figure 3:**
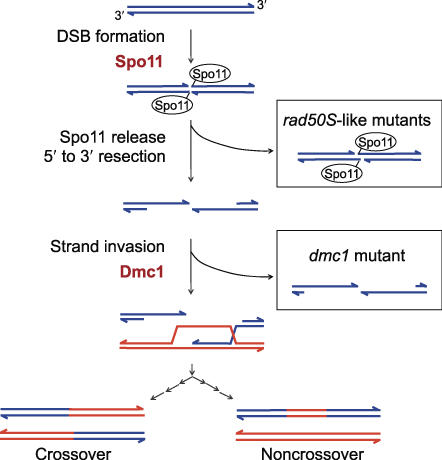
Meiotic Recombination Pathway as Elucidated in S. cerevisiae Recombination is initiated by Spo11-generated DSBs. (The DNA duplex from only one of two sister chromatids is shown.) Spo11 is then released and the 5′ ends of DSBs are degraded to yield 3′ single-stranded DNA tails. The single-stranded DNA invades one of the two sister chromatids of the homologous chromosome (only one of the homolog's chromatids is shown). Strand invasion is catalyzed by Dmc1 (and/or Rad51, not shown). Completion of repair yields either reciprocal exchange of chromosome arms that flank the break (a crossover) or no exchange of flanking arms (a noncrossover). In *rad50S*-like mutants, Spo11 remains covalently attached to DNA, and the DSBs are not repaired. In *dmc1* mutants, DSBs persist with long, single-stranded DNA tails.

Meiotic recombination occurs through the repair of programmed DNA double-strand breaks (DSBs), a process best understood in S. cerevisiae [8, 9] (Figure 3). DSBs are created by Spo11, a conserved protein that is related to an archaeal topoisomerase. When Spo11 cleaves the DNA, Spo11 simultaneously becomes covalently linked to the DSB ends. Spo11 is released from ends of the DSBs, which are then processed by exonuclease(s) to yield 3′ single-stranded tails that are substrates for strand exchange proteins such as Dmc1. Completion of DSB repair results in either reciprocal exchange of chromosome arms flanking the break (a crossover) or no exchange (a noncrossover) (Figure 3). Only crossovers physically connect homologous chromosomes for purposes of segregation at the first division, but both recombination events involve local exchange of genetic information at the DSB site.

Recombination hot spots are the sites where Spo11 preferentially forms DSBs [5]. Thus, understanding the recombination landscape requires detailed knowledge of DSB distributions. The first DSB hot spots were uncovered well over a decade ago [10–12]. Most natural hot spots in yeast are located in promoter regions of genes, with DSBs clustering within 50–200 base pairs. A locally open chromatin structure appears important, and sequence-specific DNA binding proteins are required for at least some hot spots [5]. To this day, however, molecular mechanisms dictating hot spot identity are not well understood.

## Early Cartography of DSB Distributions

Characterization of the first few DSB hot spots yielded valuable insight about the mechanism of meiotic recombination, but these studies were limited to anecdotal examples that provided limited coverage of the genome. Most of the recombination landscape was still “terra incognita”. To more fully understand recombination distributions, it was essential to obtain a more global map.

When large-scale sequence information first became available for S. cerevisiae, Baudat and Nicolas took advantage of it to carry out a systematic survey across all of chromosome III, using Southern blot analysis to map and quantify DSB hot spots [13]. A critical feature of this and other studies of DSB hot spots was the use of *rad50S*-like mutants (*rad50S*, *mre11S*, and *sae2*), in which Spo11 remains covalently attached to DSB ends [14,15] (Figure 3). Unlike the transient DSBs that are promptly repaired in wild-type cells, DSBs in *rad50S*-like mutants persist, greatly facilitating their detection and quantification.

Seventy-six hot spots were identified, almost all in intergenic, promoter-containing regions [13]. Importantly, the DSB distribution was uneven: most DSBs clustered into two large (50–100 kb) domains, one on each chromosome arm, and both telomeric regions and one centromere-proximal region were suppressed for DSB formation. This manual survey of DSBs was a tremendous effort, but chromosome III is one of the smallest in yeast (only ∼300 kb long), reflecting only ∼2.6% of the genome. The ability to extend the map to the entire genome came with the dawn of microarray technology.

Gerton and colleagues were the first to use microarrays to map global DSB distributions in yeast [16]. They also took advantage of a *rad50S*-like mutant, purifying DNA fragments covalently attached to protein (mostly Spo11) and hybridizing the protein-associated DNA to microarrays containing all 6,200 budding yeast open reading frames. DNA sequences that were highly enriched in the purified fraction relative to total genomic DNA were defined as hot spots. The results for chromosome III agreed well with the manually derived map. Moreover, the general pattern for this chromosome held true across the genome: each chromosome could be divided into hot and cold domains, with regions near centromeres and telomeres almost always low for DSB formation. The same pattern was further confirmed by numerous subsequent microarray studies, all based on enrichment of Spo11-bound DNA in *rad50S*-like mutants [17–20]. Similar methodology has also been used to map global DSB distributions in the fission yeast Schizosaccharomyces pombe [21]. Preferential formation of DSBs within discrete hot spots is also seen in this organism, although the overall organization is somewhat different, with hot spots more widely separated by genomic segments with little or no detectable DSBs.

## Problems with *rad50S*-Based Maps, and New Methods for More Accurate Mapping

Even as these mapping efforts were yielding important new insights, a growing body of evidence began to reveal that the indispensable mapmaker's tool was itself a source of inaccuracy: the *rad50S*-like mutations made it easier to measure DSBs, but at the cost of altering DSB distributions relative to wild type. One of the first hints came from the observation that *rad50S* and *dmc1* mutants gave different DSB patterns when analyzed by pulsed-field gel electrophoresis [22]. The recombination pathway is blocked at a later step in *dmc1* mutants, such that Spo11 has been removed from DSB ends and long single-stranded tails have formed (Figure 3). Another hint came from the recognition that there were not enough DSBs in *rad50S*-like mutants to account for all of the recombination in wild-type meiosis, especially near the centromere of chromosome III [13]. Chromatin immunoprecipitation experiments demonstrated that significant amounts of Dmc1 bound to this part of chromosome III in wild-type meiosis, implying that this region does indeed undergo DSB formation in normal cells, even if few DSBs can be seen in *rad50S*-like mutants [23]. Finally, direct analysis demonstrated that DSB formation is specifically repressed by *rad50S*-like mutations in regions made late-replicating by deletion of replication origins or proximity to a telomere [24]. Although the molecular basis of altered DSB distributions remains unknown, the implication was clear—large areas of the recombination landscape cannot be accurately mapped in these mutants.

A way around this difficulty is offered by new studies from the Lichten and Hochwagen groups, one published in this issue of *PLoS Biology* [25,26]. By adapting long-established methods for purifying single-stranded DNA by chromatography on benzoylated naphthoylated DEAE-cellulose, these groups fractionated meiotic DNA preparations from *dmc1* mutants to enrich for single-stranded DNA, then hybridized this material to whole-genome microarrays. Essentially all of the hybridization signal was dependent on Spo11 and thus reveals the location and amount of meiotic DSBs.

These studies markedly revise the global DSB map for yeast. One major change is the documentation of substantial DSB formation in many regions where DSBs were absent or greatly reduced in *rad50S*-like mutants, notably around centromeres and telomeres [25,26]. DSB formation is still sometimes reduced in these areas relative to hotter regions located in the arms of chromosomes, but the highly repressed zones are much smaller than previously thought (e.g., DSB-cold regions occupy the most distal 20 kb or so on each chromosome end, as opposed to the most distal ∼50 kb in *rad50S*).

It is likely that the DSB distribution in normal cells is better represented by single-stranded DNA distributions in *dmc1* mutants than by DSB maps in *rad50S*-like mutants. Notably, the *dmc1*-based maps match better with meiotic recombination maps from wild-type cells, and there is close concordance between distributions of single-stranded DNA purified from *dmc1* and from wild-type strains. Moreover, for several locations where a DSB hot spot appeared in *dmc1* maps but not in *rad50S*, Southern blot analysis demonstrated that DSBs do indeed occur in wild-type cells. Overall, more DSBs are observed in *dmc1* than in *rad50S*. Finally, whereas DSBs are under-represented in *rad50S*-like mutants in regions where DNA replication is delayed by deletion of replication origins, no such DSB suppression is seen in *dmc1* mutants [26]. Other interesting aspects of meiotic recombination are also revealed, including an apparent zone of preferential DSB formation, ∼100 kb from chromosome ends observed by Blitzblau et al., which is suggested to account for overall higher recombination rates on smaller chromosomes [25] (although this pattern was not observed in the other study; C. Buhler and M. Lichten, personal communication).

## What Is Left To Explore?

One implication of the new maps is that caution is warranted when using *rad50S*-like mutants, including in organisms other than S. cerevisiae. Another implication is that the current results may encourage similar studies in mice, where DSBs are not readily detected due to the low frequency of recombination events. Mouse mutants defective for *Dmc1* are available [27,28], but mutations fully equivalent to yeast *rad50S* are not [29]. Because mapping of meiotic single-stranded DNA could be done in wild-type yeast as well, the approach may even be applicable without *dmc1* mutations, including in humans.

Mapping of global DSB patterns in budding yeast has come a long way and has revealed many interesting patterns, but challenges still remain. One challenge revolves around the ability to identify specific hot spots using current microarray methods. As with any methodology, a key issue is the signal-to-noise ratio; in this case, it is the signal from hot spots above background hybridization noise. The problem is easily illustrated by noting that overall DSB distributions were similar in different microarray studies of *rad50S*-like mutants, but each study was only able to identify ∼200–600 discrete hot spots, with only partial agreement between studies [16–20]. This number is clearly an underestimate, because there were 76 hot spots on chromosome III alone, as mapped by more sensitive Southern blot methods [13]. By using *dmc1* mutants and applying a clever background normalization method to their microarray data, Buhler et al. were able to greatly improve the picture by identifying more than 2,100 hot spots [26]. Even with these methods, however, it remains difficult to find very weak hot spots, which may be many in number and contribute substantially to genome evolution, even if they contribute only a fraction of the DSBs in any one meiosis. A related challenge will be to increase the spatial resolution of large-scale DSB mapping. Southern blot analysis has shown that DSBs within a hot spot occur within regions 50–200 bp long. Even if the elements in a microarray were spaced closely enough, DSBs in a *dmc1* mutant cannot be mapped at such high resolution because of the large size of the single-stranded DNA track at each DSB (≥1 kb). Development of novel approaches to DSB mapping will be necessary to solve this issue.

An even bigger challenge is to understand the molecular mechanisms that shape the recombination landscape, both the larger hot and cold domains and the more highly localized hot spots within these domains. Continuing to refine the maps of this landscape helps to frame hypothesis-driven functional experiments to test these mechanisms. The latest advances in molecular mapmaking are an important step in this direction.

## Abbreviations

DSB: double-strand break
